# Photobiomodulation has rejuvenating effects on aged bone marrow mesenchymal stem cells

**DOI:** 10.1038/s41598-021-92584-3

**Published:** 2021-06-22

**Authors:** Binnur Eroglu, Evan Genova, Quanguang Zhang, Yun Su, Xingming Shi, Carlos Isales, Ali Eroglu

**Affiliations:** 1 Department of Neuroscience and Regenerative Medicine, Medical College of Georgia, Augusta University, 1120 15th Street, CA-2004, Augusta, GA 30912 USA; 2 Department of Medicine, Medical College of Georgia, Augusta University, Augusta, GA 30912 USA; 3 Department of Obstetrics and Gynecology, Medical College of Georgia, Augusta University, Augusta, GA 30912 USA

**Keywords:** Stem-cell research, Ageing, Mesenchymal stem cells

## Abstract

The plasticity and proliferative capacity of stem cells decrease with aging, compromising their tissue regenerative potential and therapeutic applications. This decline is directly linked to mitochondrial dysfunction. Here, we present an effective strategy to reverse aging of mouse bone marrow mesenchymal stem cells (BM-MSCs) by restoring their mitochondrial functionality using photobiomodulation (PBM) therapy. Following the characterization of young and aged MSCs, our results show that a near-infrared PBM treatment delivering 3 J/cm^2^ is the most effective modality for improving mitochondrial functionality and aging markers. Furthermore, our results unveil that young and aged MSCs respond differently to the same modality of PBM: whereas the beneficial effect of a single PBM treatment dissipates within 7 h in aged stem cells, it is lasting in young ones. Nevertheless, by applying three consecutive treatments at 24-h intervals, we were able to obtain a lasting rejuvenating effect on aged MSCs. Our findings are of particular significance for improving autologous stem cell transplantation in older individuals who need such therapies most.

## Introduction

Mesenchymal stem cells (MSCs), also called stromal cells, originate from the mesoderm germ layer^1^ and are known to be highly plastic with the ability to differentiate into a variety of cell lineages, including osteogenic, chrondrogenic, adipogenic, myogenic, and neurogenic lines^2–4^. Therefore, MSCs hold great promise in a myriad of medical fields—including regenerative medicine, tissue engineering, and the treatment of both chronic and acute diseases—with therapeutic benefits of MSCs having already been demonstrated for use in acute myocardial infarction, peripheral vascular disease, bony tissue defects, recurrent Crohn’s fistulae, and chronic skin wounds^5–11^. However, the functional capacity of stem cells has been shown to decline with aging^12–15^. In particular, MSCs from older donors exhibited both diminished plasticity and proliferation ability compared to ones isolated from younger donors^12–14^. Moreover, transplanted BM-MSCs isolated from older mice (1-year old) seem to be unable to efficiently populate and differentiate into somatic cells compared to ones isolated from young mice^14^. Consequently, the autologous transplantation of MSCs is expected to be inefficient in older subjects who most need MSC transplantation. The aforementioned deficiencies of aged MSCs have been linked to mitochondrial dysfunction^16–18^. The role of mitochondrial dysfunction in aging has been well demonstrated in the POLG knock-in mouse model that is characterized by premature aging and reduced lifespan as a result of mutated DNA polymerase gamma (POLG), which controls replication of mitochondrial DNA (mtDNA)^19,20^. Moreover, transplantation of stem cells from POLG knock-in mice to non-mutated normal recipients recapitulated the premature aging features of the POLG knock-in model^21^.

In addition to being the powerhouse of the cell, mitochondria play a critical role in numerous cellular events, including cell signaling, cell specialization and growth, apoptosis, and senescence^18^. As both the major production and scavenging site of reactive oxygen species (ROS), mitochondria maintain a tight balance between the two; this balance is critical for ROS-dependent physiologic signaling and for avoiding free-radical damage that leads to cell death. Aging-associated stresses such as chronic inflammation and extended exposure to environmental toxins compromise normal function of mitochondria, resulting in imbalanced ROS generation that, in turn, starts a vicious cycle of increased mitochondrial disfunction and thus free radical production. Taken together, mitochondria represent an important target for mitigation of the functional decline of aging stem cells.

Photobiomodulation (PBM), also known as low-level laser therapy (LLLT), offers a potential way to directly improve mitochondrial function^22–24^. PBM relies on the presence of chromophores, several of which exist in the mitochondrial membrane^24,25^. By absorbing photons, these chromophores can induce different signaling pathways, and thus allow light to influence overall mitochondria and cell behavior^26^. In particular, Cytochrome C Oxidase (CcO), an enzyme in the electron transport chain involved in adenosine triphosphate (ATP) production, exhibits peak absorbance at 830 nm^27^, and wavelengths in this near-infrared (NIR) range have shown promise in the field of PBM^28^. While other wavelengths have also been studied, they appear less effective at improving mitochondrial activity and can actually inhibit mitochondrial functionality^24,29^. Stem cells exposed to NIR light exhibit increased proliferation^30,31^ and additionally have a greater mitochondrial membrane potential (MMP) and improved ATP generation^24,32^, with effects being most prominent between 3 and 6 h after treatment^33^.

Despite the connection between aging and mitochondrial decline, much of the research that currently exists on PBM focuses on mechanism or its usefulness for wound-healing and reducing pain, inflammation, and edema^24,34^; The objective of the present study was to fill this gap and improve stem cell therapies—especially the efficacy of autologous stem cell transplantation in older individuals—by rejuvenating stem cells through improvement of their mitochondrial functionality. To test the hypothesis that overall effectiveness of aged stem cells can be restored to the level of young stem cells by PBM, MSCs isolated from bone marrow of both young (3-month-old, 3 m) and old (24-month-old, 24 m) C57BL/6 mice were subjected to different PBM regimens and their proliferation, oxygen consumption rate (OCR), and ATP production were evaluated with respect to those of untreated counterparts, as was expression of senescence/juvenescence markers (i.e., p21, p16, Nrf2, and Sirt1). The findings of the present study show in particular that consecutive PBM treatments have a lasting rejuvenating effect on aged MSCs.

## Materials and methods

### Experimental overview

**Figure 1:**
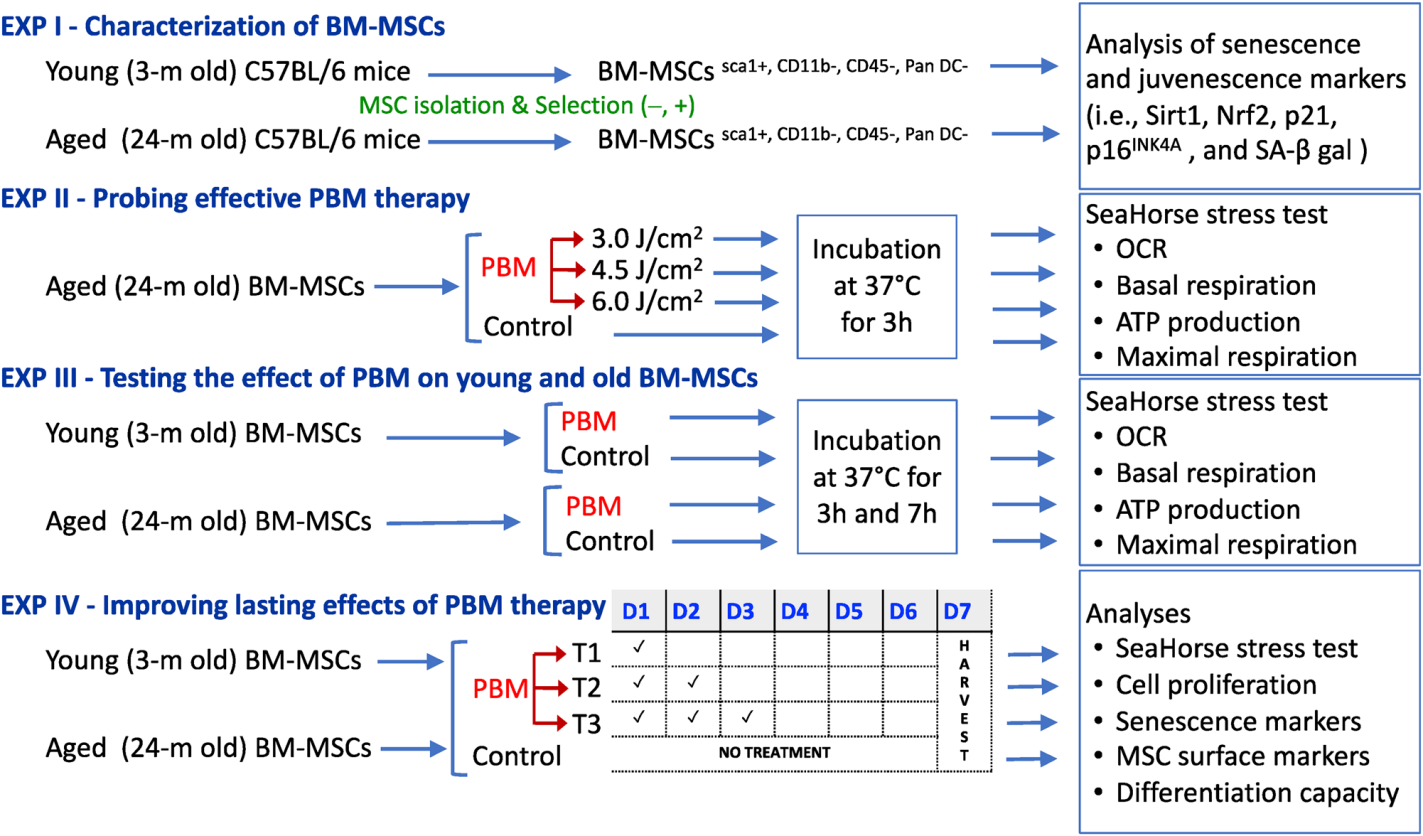
Experimental overview. A total of four sets experiments were performed with different subgroups. Bone marrow mesenchymal stem cells (BM-MSCs) isolated from young and old mice underwent different treatments and were analyzed at the end of each experimental repeat as shown.

The first set of experiments involved characterization of bone-marrow derived mesenchymal stem cells (BM-MSCs) isolated from young (3 m) and old (24 m) mice (Fig. 1). The second and third sets of experiments were designed to find an effective PBM treatment for aged BM-MSCs and to compare responses of young and aged BM-MSCs to an optimized PBM treatment at two different time points, respectively. The final set of experiments aimed at reversing aging of MSCs (Fig. 1). Overall, this study was carried out in compliance with the ARRIVE guidelines.

### Reagents and media

All the reagents and media were purchased from ThermoFisher unless otherwise stated. Agilent Seahorse XF DMEM medium, pH 7.4 (#103575-100), Seahorse XFe96 FluxPacks (contain XFe96 sensor cartridges, XF96 culture microplates, and XF Calibrant), and Seahorse XF Cell Mito Stress Test kit (#103015-100) were purchased from Agilent Technologies (Santa Clara, CA). d-(+)-Glucose (#G7021), l-Glutamine (#G8540), biotin (#B4639), human insulin (#91077C), and Cell Proliferation Reagent WST-1 (#5015944001) were purchased from Sigma. Alizarin Red (#22889), Oil Red-O (#01794), d-pantothenate (#00047), and β-glycerophosphate (#01454) were purchased from Chem-Impex International.

### Isolation and culture of mesenchymal stem cells

All protocols for isolation of BM-MSCs were approved by the Institutional Animal Care and Use Committee (IACUC) at Augusta University (Protocol # 2008-0302) and all animal procedures were performed in accordance with the IACUC guidelines and regulations. BM-MSCs were extracted from young (3 m) and aged (24 m) C57BL/6 mice as described elsewhere^35,36^. Briefly, six mice per age group were euthanized with CO_2_ followed by thoracotomy according to the IACUC guidelines. Subsequently, femora and tibiae were dissected and cut open at both ends. Bone marrows were then flushed with RPMI-1640 containing 9% fetal bovine serum (FBS), 9% horse serum, 100 U/ml penicillin, 100 µg/ml streptomycin, and 12 µM l-glutamine using a 22-gauge syringe needle. The flushed bone marrows were filtered through a 70-µm nylon filter, and then further dispersed by passing through a 25-gauge syringe needle. The resulting single cell suspension in the flushing medium was distributed to 175-cm^2^ flasks at a density of 2 × 10^7^ cells/flask for a 3-h incubation period at 37 °C under 5% CO_2_. At the end of the incubation period, nonadherent cells were removed by aspirating the media and gently washing each flask twice. Once adherent cells reached ~ 80% confluency, BM-MSCs were first isolated using negative immune-depletion (magnetic beads conjugated with anti-mouse CD11b, CD45R/B220, and Pan DC) and then positive immune-selection (anti-Sca-1 beads) as described previously^35^.

The mouse MSCs were cultured in KnockOut Dulbecco’s modified Eagle’s medium: Nutrient Mixture F-12 (DMEM/F-12) supplemented with 10% FBS, 1 × glutamax (2 mM l-alanine-l-glutamine), and 1% antibiotic/antimycotic mix. The culture medium was partially replaced every other day, and the cultures were incubated in a humidified incubator at 37 °C under 5% CO_2_ in air. Once the culture reached approximately 80% confluence, the cells were washed with Versene and subsequently detached from the plate using 0.25% Trypsin–EDTA. After inhibiting trypsin by adding serum-containing medium in 1:1 ratio, the cells were centrifuged at 280×*g* for 10 min and the cell viability was determined by trypan blue exclusion test upon resuspension in the culture medium.

### PBM treatment

MSCs from young (3 m) and aged (24 m) mice were treated with a diode laser (Diode IR Laser System, 808M100, Dragon Lasers) with continuous wave at 808-nm as described before^37^. The experimental groups were exposed to 16.66, 25.00, or 33.33 mW/cm^2^ of 808-nm light for 180 s, resulting in 3.0, 4.5, and 6.0 J/cm^2^ of total energy being delivered to the cells respectively. The dose (expressed as J/cm^2^) was calculated by total irradiation time in seconds × power output (mW/cm^2^)/1000. For Seahorse extracellular flux analysis and cell proliferation experiments, the cells were exposed to 3-J/cm^2^ PBM once, twice, or thrice at 24-h intervals. The irradiation process was performed at room temperature. The cells were incubated in a humidified incubator at 37 °C under 5% CO_2_ between the exposures. Untreated cells without any laser exposure served as controls.

### Agilent Seahorse XF Cell Mito Stress test and oxygen consumption rate

The day before the assay, the Seahorse XF sensor cartridge was hydrated with water and kept in a non-CO_2_ humidified incubator at 37 °C. Seahorse XF Calibrant solution was also kept in a non-CO_2_ incubator at 37 °C.

On the day of analysis, both young and aged BM-MSCs at the same passage were harvested, counted, and an equal number of cells (7500/well) was seeded in the XF 96-well culture plate. The four corners of the culture plate were left unseeded for background correction. The seeded cells were allowed to adhere to the wells for 1 h at room temperature, and then cultured for 2 h in a humidified incubator at 37 °C under 5% CO_2_ in air before undergoing PBM treatment_._ After the treatment, the XF 96-well culture plate was returned to the incubator and kept there until the assay’s start. Before the analysis, the culture medium was removed and the cells were washed with pre-warmed XF assay medium consisting of 10 mM glucose, 1 mM sodium pyruvate, and 2 mM glutamine (pH 7.4). For pre-equilibration, the cells were maintained in the assay medium at 37 °C in a non-CO_2_ incubator for 1 h. Meanwhile, oligomycin (1.5 μM), carbonyl-cyanide-4-(trifluoromethoxy) phenylhydrazone (FCCP, 1 μM), and rotenone/antimycin A (0.5 μM) compounds from the Seahorse XF Cell Mito Stress Test kit were prepared according to manufacturer’s instructions and loaded into the injection ports of the sensor cartridge in the order of injection.

Measurement of the oxygen consumption rate (OCR, pmol/min), an indicator of mitochondrial respiration, was performed using an XFe96 Extracellular Flux analyzer^38^. After the calibration of the sensor cartridge in the analyzer, the OCR was measured at baseline, as well as upon consecutive injections of oligomycin, FCCP, and rotenone/antimycin A. After the assay, the cells were washed with phosphate buffered saline (PBS) and lysed with radioimmunoprecipitation assay (RIPA) buffer containing 150 mM NaCl, 1% NP-40, 0.5% sodium deoxycholate, 0.1% SDS, and 50 mM Tris–HCl (pH 8.0) supplemented with 1 × protease inhibitors. Protein concentration of each well was determined using the BCA protein assay. The data was normalized to μg of protein. The Wave software (version 2.6.1) was used to analyze the data.

### Cell proliferation assay

Young (3 m) and aged (24 m) BM-MSCs were plated on a 96-well culture plate at a density of 1000 cells/well. The BM-MSCs assigned to three different experimental groups (i.e., T1, T2, and T3) were treated with a 3-J/cm^2^ energy dose of PBM once a day for 1, 2, or 3 days while the BM-MSCs in the control group did not receive any treatment (Fig. 1). On day 7, the cells were harvested from the control and experimental groups, and then the viability and total number of cells were determined in each group by trypan blue exclusion test using a hemocytometer. The cell viability in each group was normalized to the untreated young control and expressed as a relative percentage.

In a separate set of experiments, the proliferation of the control and treated cells was also assessed using Cell Proliferation Reagent WST-1 to verify the accuracy of our findings. On day 7 following the treatment with PBM 1 to 3 times as described above, the culture medium of all groups was replaced with a fresh one and Cell Proliferation Reagent WST-1 was added at a 1:10 final dilution. After 2 h of incubation at 37 °C under 5% CO_2_ in air, the absorbance was measured at 450 nm and 650 nm using a plate reader (FLUOstar Omega). The culture medium with 10% WST-1 alone was used as a blank and its reading was subtracted from all values. The cell viability in each group was then normalized to that of the untreated young controls and expressed as a relative percentage.

### Western blotting and detection of senescence markers

Irradiated and non-irradiated cells were lysed in the RIPA buffer supplemented with 1X protease inhibitors. The protein samples (10–20 μg) were electrophoresed on 10–12% SDS-PAGE and transferred to polyvinylidene fluoride (PVDF) membranes (cat#LC2002, Novex, San Diego, CA and cat#10600029, GE Healthcare). Membranes were blocked in 3% bovine serum albumin (BSA, cat#7500804, Lampire Biological Laboratories) prepared in PBS with 0.1% Tween-20 (PBST), and immunoblotted with the following senescence and juvenescence (or longevity-promoting) markers: p21, p16^INK4a^, Sirt1, and Nrf2 (cat#A1483, A0262, A0230, and A0674, respectively, ABClonal). β-actin was used as a loading control. The immunoreactive bands were visualized using Clarity enhanced chemiluminescent substrate (cat#1705060, Bio-Rad). The quantification of the western blot bands was performed using ImageJ software^39^.

### Immunofluorescence

After the cells were irradiated 1–3 times as described above, the untreated control and the irradiated cells were harvested on day 7. The cells were plated at a density of 1000 cells/well on cover glasses previously coated with collagen. After the cells were attached (3 h), they were fixed in 4% paraformaldehyde (PFA, #158127, Sigma) for 10 min and rinsed with PBS. The cells were blocked with 3% BSA in PBS containing 0.1% Triton-100 for 1 h at room temperature and incubated overnight at 4 °C with p21 (1:200 dilution), PDGFRα (#A2103), PDGFRβ (#A2180), or Vimentin (#A2584) primary antibodies (1:100 dilution, ABClonal). After washing the cells with PBS-0.1% Triton-100, they were incubated with the secondary antibody (Alexa Fluor-555 goat anti-rabbit IgG, #A-21428, and 1:500 dilution) for 1 h at room temperature. For imaging, nuclei were counterstained with Hoechst 33342 (#H1399, 5 μg/ml) and coverslips were mounted using a mounting medium. Fluorescent images were captured using a fluorescent microscope (BZ-X710, Keyence) equipped with an air objective Plan Fluor 40 ×/0.60 Ph2 (Nikon). To compare the fluorescence intensity, images were taken using the same exposure conditions. The fluorescence intensity was quantified using ImageJ software, and the corrected total cell fluorescence (CTCF) was calculated using the following formula: CTCF = integrated density − (area of selected cell × mean fluorescence of background reading).

### Senescence assays

Senescence-associated β-galactosidase (SA-β-Gal) activity was examined using a Senescence β-Galactosidase Staining Kit (Cell Signaling Technology, #9860) according to the manufacturer’s instructions. Briefly, BM-MSCs were seeded in a 24-well plate at a density of 50,000/well and were treated with a 3-J/cm^2^ energy dose of PBM for 3 days in a row (T3) as described earlier. On day 7, the untreated control and treated cells were rinsed with PBS, and then fixed with the fixative solution provided by the kit for 15 min. Next, the cells in each well were rinsed with PBS and stained with freshly prepared β-galactosidase staining solution at 37 °C in a dry incubator for 20 h. Representative images from each replicate were then acquired by a Zeiss Axiovert 40 CFL inverted microscope with a Mightex camera. The area stained with SA-β-Gal in each replicate was calculated as a percentage by ImageJ software^39^.

### Multi-lineage differentiation of BM-MSCs

Osteogenic, adipogenic, and chondrogenic differentiation of BM-MSCs were induced as previously described^40^. Briefly, BM-MSCs were seeded in a 24-well plate and were treated with a 3-J/cm^2^ energy dose of PBM for 3 days in a row (T3) as described earlier. On day 7, the cells were harvested, and an equal number of cells (200,000/well) were plated for each group. After the cells attached, the medium of both the treated and untreated control cells was replaced with a differentiation medium.

For osteogenic differentiation, BM-MSCs were cultured in the osteogenic differentiation medium (DMEM/F-12 including 10% FBS, 100 nM dexamethasone, 10 mM β-glycerophosphate, and 0.05 mM l-ascorbic acid-2-phosphate) for 18 days while replacing the medium every 3 days. The cells were then fixed in 4% PFA at room temperature for 30 min. To visualize calcium deposits, the cells were stained with 2% Alizarin Red solution (pH 4.2) at room temperature for 45 min^40^. Representative images from each replicate were acquired by a Zeiss Axiovert 40 CFL inverted microscope with a Mightex camera. The area stained with Alizarin Red of each image was calculated as a percentage using ImageJ software. The values were then normalized to that of the untreated young controls and expressed as a relative percentage.

For adipogenic differentiation, BM-MSCs were first cultured in adipogenic differentiation medium (DMEM/F-12 containing 3% FBS, 0.25 mM 1-methyl-3-isobutylxanthine [IBMX] 5 μM rosiglitazone [Adipogen, San Diego, CA], 1 μM dexamethasone, 66 μM biotin, 34 μM d-pantothenate, and 200 nM human insulin) for 3 days. The medium was then changed to adipogenic maintenance medium using the same reagents without rosiglitazone and IBMX. Following 15 days of culture in the adipogenic maintenance medium, the cells were fixed in 4% PFA, and the lipid formation was examined using Oil Red-O staining as described before^40^. Represantative images were acquired by a Zeiss Axiovert 40 CFL inverted microscope with a Mightex camera. The area stained with Oil Red-O on each image was calculated as a percentage using ImageJ software. The values were then normalized to that of the untreated young controls and expressed as a relative percentage.

For chondrogenic differentiation, BM-MSCs were pelleted in 15-ml polypropylene tubes via centrifugation at 150×*g* for 5 min. The pelleted cells were then cultured in chondrogenic induction medium (DMEM/F-12 containing 1% FBS, 100 nM dexamethasone, 10 ng/ml TGF-β1, 500 ng/ml BMP-6, 0.16 mM l-ascorbic acid-2-phosphate, 1% glutamax, 1% Insulin-Transferrin-Selenium-Ethanolamine [ITS-X], and 1% antibiotic/antimycotic) for 21 days. The medium of each group was changed every 3 days. At the end of the culture period, the pellets in each tube were fixed in 4% PFA at room temperature for 30 min, and then stained with 1% Alcian blue staining solution as described earlier^40^. Images were taken using a Zeiss Axio Vert.A1 inverted microscope equipped with AxioCam.

### Statistical analysis

Experiments were repeated at least three times, and data were presented as the mean ± standard error of mean (SEM). Quantitative data were analyzed by Student’s t-test or ANOVA using Graph Pad Prism 9.1.0 (GraphPad Software, Inc., San Diego, CA)^41^. Differences between the groups were considered significant at *p* < 0.05.

## Results

### Characterization of young and aged MSCs

**Figure 2:**
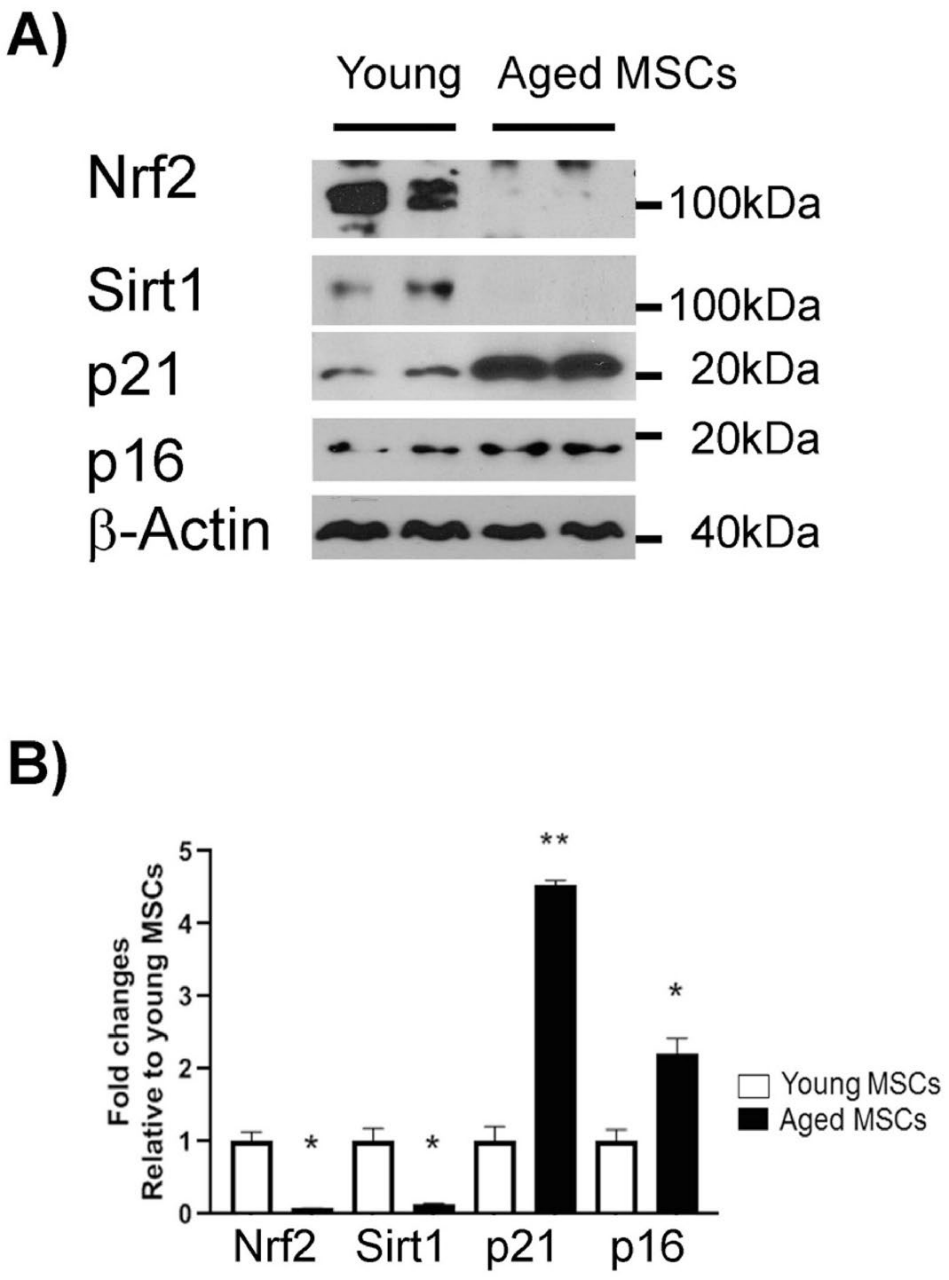
Characterization of young and aged MSCs by western blotting. Protein levels of four senescence markers were evaluated in young and aged MSCs at the same passage where β-actin was used as a loading control (**A**). The band intensities were quantified by ImageJ software (**B**). The bars represent means ± standard error of mean. *p < 0.05; **p < 0.01.

Bone marrows aspirated from femora and tibiae of young (3 m) and old (24 m) C57BL/6 mice were subjected to negative (CD11b, CD45, and Pan DC) and positive (Sca1) selections to isolate MSCs as described elsewhere^35^. Subsequently, the multilineage differentiation capacity (i.e., differentiation into osteogenic, chondrogenic, and adipogenic lineages) of the isolated cells was examined and confirmed as described^40^. To further characterize the selected cell populations in terms of aging, the resulting BM-MSCs^sca1+, CD11b −, CD45 −, Pan DC −^ were subsequently analyzed for the expression of senescence and longevity-promoting markers by immunoblotting (Fig. 2). Previous studies have shown that Sirtuin 1 (Sirt1) plays a critical role in longevity^42–45^, and its expression and activity are decreased in aged cells, including MSCs^46–48^. Similarly, Nuclear factor erythroid 2-related factor 2 (Nrf2) has been shown to have a clear longevity-promoting effect^49,50^, and its expression is decreased with age^51–53^. In contrast, senescence-associated cell cycle arrest markers p21 and p16^INK4A^ are increasingly expressed by aging and commonly used for testing cellular senescence^54–57^. Therefore, we analyzed the level of these four markers in young and aged BM-MSCs by western blotting using β﻿-actin as a reference. Our immunoblot data showed that compared to the young BM-MSCs, the aged BM-MSCs exhibited 7.2- and 12.5-fold decrease in expressions of Sirt1 and Nrf2, respectively, whereas the expression of p21 and p16^INK4A^ was increased by 4.5- and 2.2-fold, respectively (Fig. 2). These findings indicate that BM-MSCs from old mice are indeed different than the ones from young mice and display a marked senescence phenotype. Based on the expression profiling data, we used these cells in subsequent experiments.

### Probing effective PBM therapy dose

**Figure 3:**
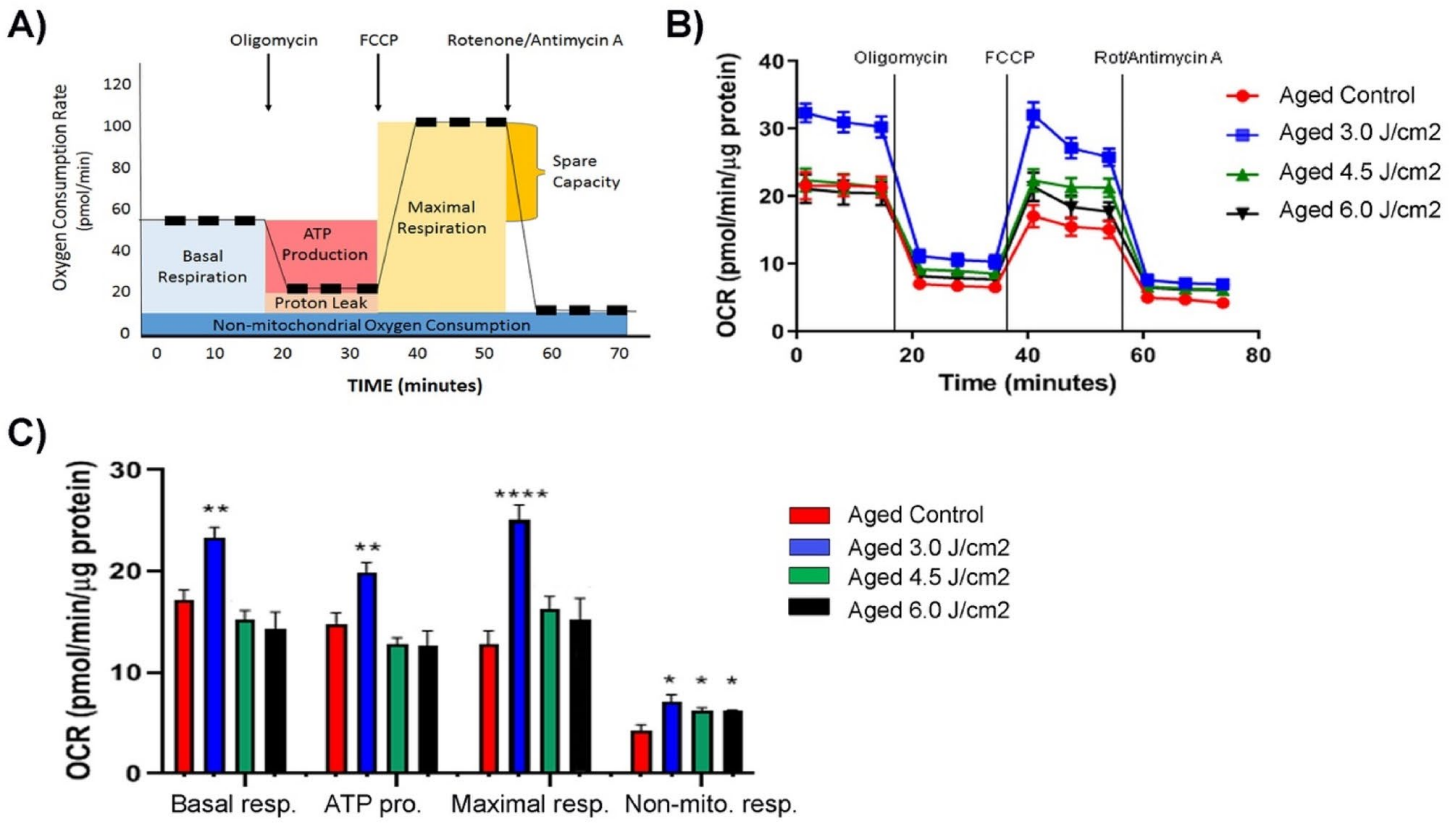
Effects of different PBM doses on mitochondrial function of aged MSCs. (**A**) Shown is a schematic representation of Agilent Seahorse OCR (oxygen consumption rate) curve and the key parameters of mitochondrial respiration. Injection points of Oligomycin, FCCP, and Rotenone/Antimycin A are indicated by arrows. (**B**) Mitochondrial response of aged MSCs to three different PBM doses. Basal respiration, ATP production, maximal respiration, and non-mitochondrial respiration of aged MSCs were determined 3 h after PBM treatment using the Seahorse Mito Stress Test kit, with untreated MSCs serving as controls. Data were normalized to μg of protein. (**C**) Plotted are the kinetic data on basal respiration, ATP production, maximal respiration, and non-mitochondrial respiration as a bar graph and shown are significant differences among the controls and treatment groups. The bars represent means ± standard error of mean. *p < 0.05; **p < 0.01; ****p < 0.0001.

To find an effective PBM dose for aged MSCs, we used an NIR wavelength (808 nm) and examined the effect of three different energy densities (i.e., 3.0, 4.5, and 6.0 J/cm^2^) on aged BM-MSCs. Untreated aged BM-MSCs served as controls. Three hours after the treatment, the oxygen consumption rate (OCR) of the cells was determined using the Seahorse metabolic flux analyzer. The basal respiration and the respirations after consecutive injections of oligomycin, FCCP, and rotenone/antimycin A are shown in Fig. 3A. The basal respiration reached the highest level when aged MSCs (isolated from 24 m mice) were treated with an energy density of 3.0 J/cm^2^ (Fig. 3B). This increase was statistically significant compared to two other dosages and the control group (*p* < 0.01) (Fig. 3C). Following oligomycin treatment, which reflects respiration coupled to ATP production, the production of ATP was highest after the 3.0 J/cm^2^ dose as compared to the control (*p* < 0.01). Following FCCP treatment, which increases OCR to its maximum level, the maximal respiratory capacity was significantly increased in cells treated with 3.0 J/cm^2^ (*p* < 0.0001) compared to control cells. No significant changes were observed between the cells treated with either 4.5 J/cm^2^ or 6.0 J/cm^2^ and control cells. Taken together, these results indicate that the PBM treatment delivering an energy density of 3.0 J/cm^2^ is the best modality for aged MSCs among the tested conditions and was used for subsequent experiments.

### Effects of PBM therapy on young and aged MSCs

**Figure 4:**
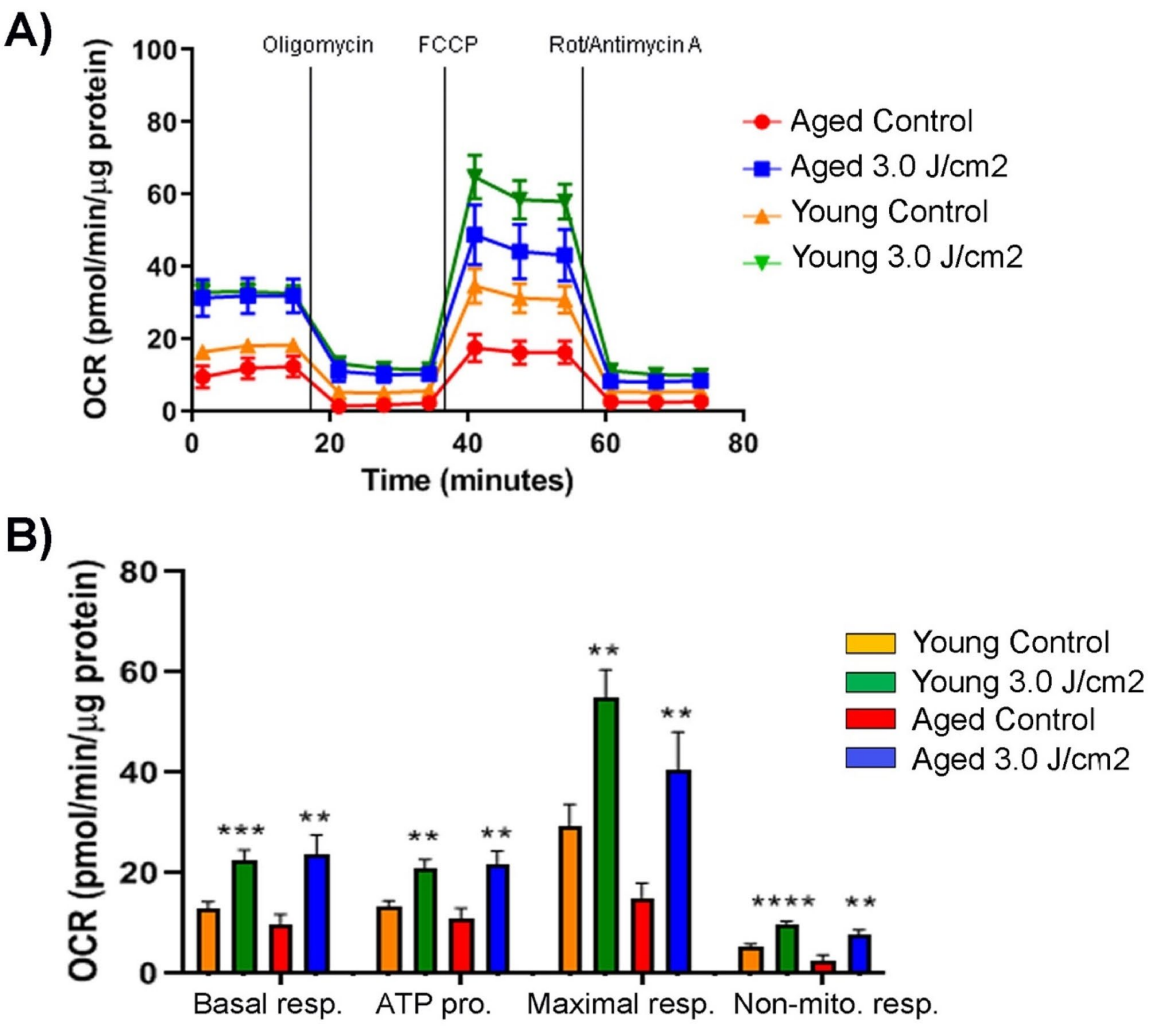
Comparison of short-term effects of PBM on young and aged MSCs. Both young and aged MSCs underwent a PBM treatment at 3 J/cm^2^ and their mitochondrial response was determined 3 h later along with that of untreated controls. Data were normalized to μg of protein. Shown are basal respiration, ATP production, maximal respiration, and non-mitochondrial respiration of young and aged MSCs as an OCR curve (**A**) and as a bar graph (**B**) with significant differences labeled. The bars represent means ± standard error of mean. **p < 0.01; ***p < 0.001; ****p < 0.0001.

Upon determining an effective PBM dosage, both young and aged MSCs were treated with that (i.e., 3.0 J/cm^2^ using 808 nm wavelength) and mitochondrial respiration was measured 3 h after the treatment. As shown in Fig. 4, the PBM treatment at 3.0 J/cm^2^ significantly increased mitochondrial respiration in both young and aged MSCs compared to their untreated counterparts. Interestingly, basal respiration, ATP production, maximal respiration, and non-mitochondrial respiration of the treated aged MSCs were higher than those of untreated young MSCs and were statistically comparable to those of treated young MSC. Overall, these results suggest that a proper PBM treatment restores the mitochondrial respiration of aged MSCs to the level of young cells.

**Figure 5:**
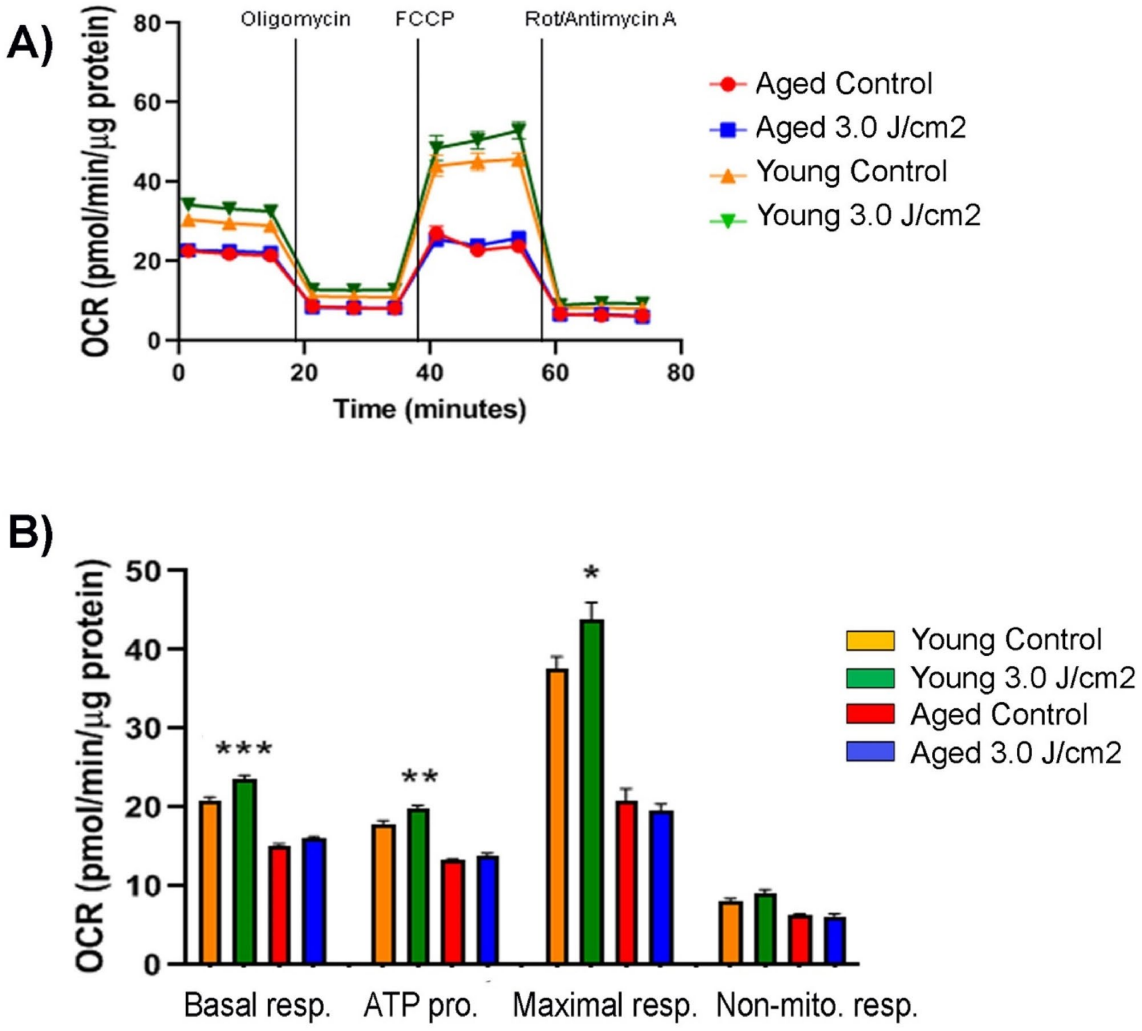
Comparison of lasting effects of PBM on young and aged MSCs. Both young and aged MSCs underwent a PBM treatment at 3 J/cm^2^ and their mitochondrial response was determined 7 h later along with that of untreated controls. Data were normalized to μg of protein. Shown are basal respiration, ATP production, maximal respiration, and non-mitochondrial respiration of young and aged MSCs as an OCR curve (**A**) and as a bar graph (**B**) with significant differences labeled. The bars represent means ± standard error of mean. *p < 0.05; **p < 0.01; ***p < 0.001.

To address if the beneficial effect of PBM therapy on young and aged MSCs persists beyond 3 h, we next repeated the same PBM treatment at 3.0 J/cm^2^, but performed the Seahorse XF Mito Stress Test 7 h after the treatment (Fig. 5A). Compared to untreated controls, basal respiration, ATP production, and maximal respiration of treated young MSCs remained significantly higher at 7 h posttreatment (Fig. 5B). In contrast, none of the examined parameters in treated aged MSCs were significantly better than those of untreated counterparts. These data indicate that the beneficial effect of PBM therapy lasts longer in young MSCs and still remains significant at 7 h following the treatment, yet completely disappears in aged MSCs.

### Improving lasting effects of PBM therapy

**Figure 6:**
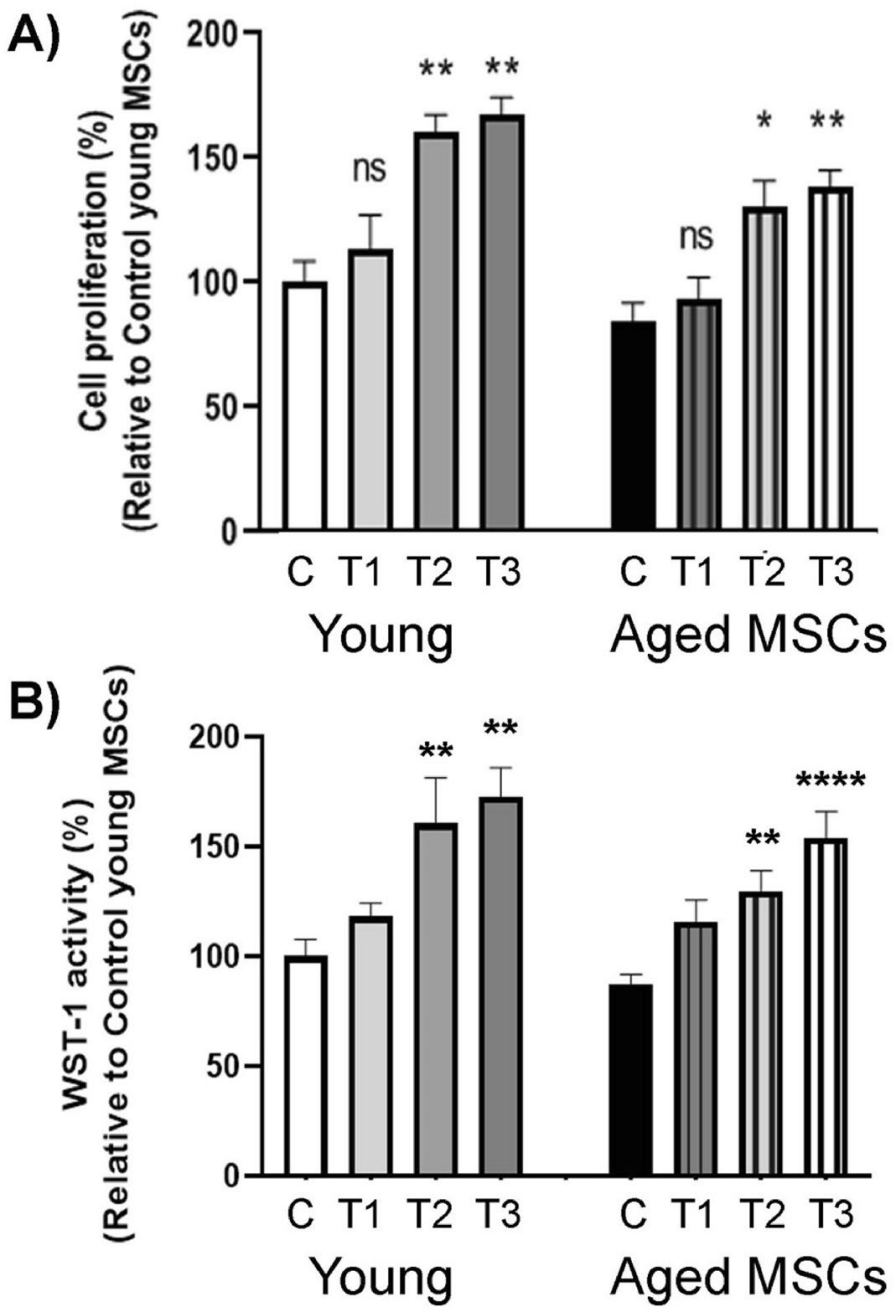
Effects of single and multiple doses of PBM on proliferation of young and aged MSCs: Both young and aged MSCs underwent a PBM treatment at 3 J/cm^2^ for 1, 2 or 3 days in a row. The control group did not receive any treatment. (**A**) On day 7, the cells from both the control and the experimental groups were harvested to determine the cell viability and total number of cells in each group using a trypan blue exclusion assay. Shown is the percentage of cell proliferation in each group relative to the untreated young MSC control group, with significant differences indicated by asterisk signs. *p < 0.05; **p < 0.01. (**B**) On day 7, metabolic activity was measured using WST-1 assays. Shown is the percentage of cell proliferation in each group relative to the untreated young MSC control group with significant differences indicated by asterisk signs. **p < 0.01; ****p < 0.0001.

To test our working hypothesis that consecutive PBM treatments at 24-h intervals induce a lasting rejuvenating effect on aged MSCs, we first subjected both young and aged MSCs to increasing numbers of PBM treatment sessions, and then investigated the resulting effect on cell proliferation on day 7 (Fig. 6). For this purpose, both young and aged MSCs were seeded in a 96-well plate in quadruplets, and the cells in the experimental groups were treated with a daily 3-J/cm^2^ PBM dose for 1, 2 or 3 consecutive days, corresponding to the experimental groups T1, T2, and T3, respectively. When the proliferation of young and aged MSCs in each treatment group was evaluated on day 7 with respect to untreated young (100%) and aged controls (85%), the single treatment slightly improved proliferation of both young (114%) and aged MSCs (94%) but these improvements were not statistically significant (Fig. 6A). In contrast, two and three consecutive treatments significantly improved proliferation of both young (161% and 168%, respectively) and aged (131% and 139%, respectively) MSCs compared to untreated control cells (Fig. 6A). To confirm our findings by another test, the proliferation of the treated and untreated control MSCs was also evaluated by the WST-1 assay. When the WST-1 activity of young and aged MSCs in each treatment group was evaluated on day 7, we found an insignificant improvement after the single treatment for young (100% vs. 118%) and aged (87% vs. 116%) MSCs (Fig. 6B). By contrast, two and three consecutive treatments yielded significantly improved WST-1 activity for both young (161% and 173%, respectively) and aged (130% and 154%, respectively) MSCs compared to untreated control cells (Fig. 6B). Together, these data suggest a lasting beneficial effect from consecutive PBM treatments.

**Figure 7:**
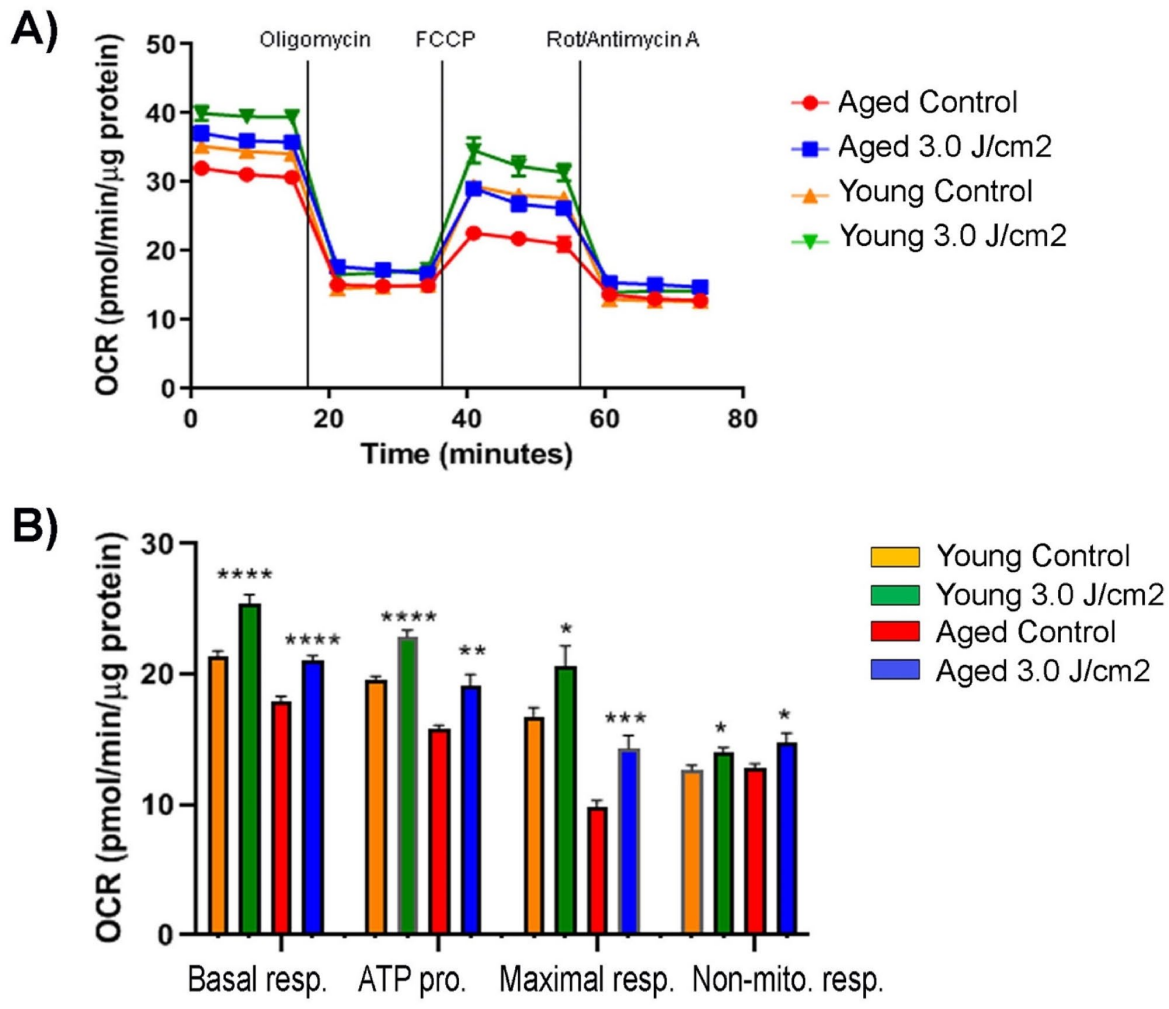
Effects of multiple doses of PBM on mitochondrial function of young and aged MSCs. Both young and aged MSCs underwent a PBM treatment at 3 J/cm^2^ for three consecutive days, whereas the young and aged MSC control groups did not receive any treatment. Seven hours after the last treatment, OCR was measured under basal conditions and following sequential injections of oligomycin, FCCP, and rotenone/antimycin A compounds. Data were normalized to μg of protein. Shown are basal respiration, ATP production, maximal respiration, and non-mitochondrial respiration of young and aged MSCs as an OCR curve (**A**) and as a bar graph (**B**), with significant differences indicated by asterisk signs. The bars represent means ± standard error of mean. *p < 0.05; **p < 0.01; ***p < 0.001; ****p < 0.0001.

Next, we investigated the effect of the multiple doses of PBM therapy applied for 3 consecutive days on mitochondrial respiration. After plating on a 96-well plate, both young and aged MSCs were treated with an energy density of 3 J/cm^2^ once a day, for 3 days in a row, and mitochondrial respiration rates were measured 7 h later after the last treatment (Fig. 7A). Untreated young and aged MSCs served as controls. As shown in Fig. 7B, all the examined parameters were significantly improved in both young and aged MSCs following three successive treatments with 24-h intervals, indicating the lasting beneficial effect.

**Figure 8:**
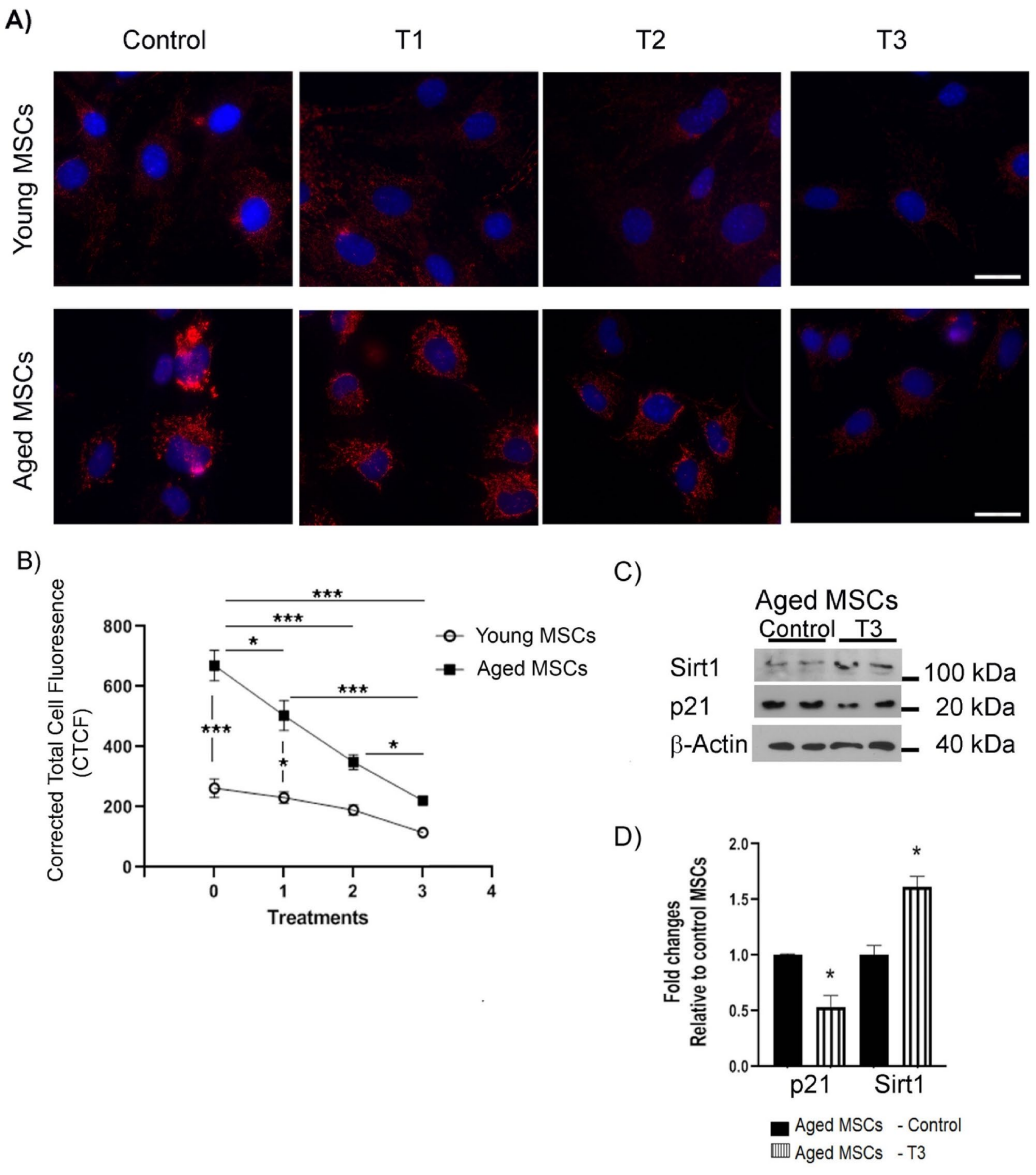
Effects of multiple doses of PBM on senescence marker levels of young and aged MSCs: Young and aged MSCs underwent a PBM treatment at 3 J/cm^2^ for 1 (T1), 2 (T2) or 3 (T3) days in a row, while untreated MSCs served as controls. On day 7, the cells from both the control and experimental groups were harvested and subjected to either immunofluorescence staining or immunoblotting to evaluate their p21 and Sirt1 levels. (**A**) Shown are representative immunofluorescence images stained for p21 (red) and cell nuclei (blue). Scale bars: 20 μm. (**B**) Shown are corrected total cell fluorescence (CTCF) for each group after quantification of p21 fluorescence intensity using ImageJ. The error bars represent the standard error of mean. (**C**) Immunoblot analysis of p21 and Sirt1 in aged MSCs that were either treated with a 3-J/cm^2^ fluence for 3 consecutive days (T3) or remained untreated (Control). β-actin was used as a loading control. (**D**) p21 and Sirt1 levels obtained by quantification of immunoblot bands and plotted as a bar graph, with significant differences indicated by asterisk signs. The bars represent means ± standard error of mean. *p < 0.05; ***p < 0.001.

To further assess how senescence markers are affected after daily PBM treatment for 3 consecutive days, we performed both immunofluorescence and immunoblot experiments (Fig. 8). In the first experiment, both young and aged MSCs were either remained untreated (controls) or exposed to a 3-J/cm^2^ dosage of PBM in three treatment groups (T1, T2, and T3) for once, twice, or thrice with 24-h intervals (see Fig. 1 for the experimental design). On Day 7, MSCs in the control and treatment groups were fixed and stained for the senescence marker p21 (Fig. 8A). Consistent with the initial results (Fig. 2), untreated aged MSCs displayed more than twofold higher fluorescence intensity compare to untreated young MSCs (*p* < 0.05, Fig. 8B). Importantly, immunofluorescence staining of p21 was increasingly diminished in both young and aged MSCs with each consecutive PBM treatment (Fig. 8B). As the initial p21 fluorescence intensity was almost at the basal level in the untreated young MSC control group, the PBM-induced decrease in the p21 fluorescence intensity was modest in treated young MSCs and did not reach a significant level compared to their untreated controls even after 3 consecutive PBM treatments (T3). In contrast, each PBM treatment significantly diminished the p21 fluorescence intensity in aged MSCs compared to their untreated counterparts, suggesting rejuvenating effect of PBM. In fact, aged MSCs subjected 3 consecutive treatments had a p21 fluorescence intensity similar to that of young MSCs (Fig. 8B).

In the subsequent experiment, aged MSCs underwent three consecutive PBM treatments at 3 J/cm^2^ similar to previous experiments and were analyzed on Day 7 for p21 and Sirt1 using immunoblotting whereas untreated aged MSCs served as controls. Our western blot analysis revealed that compared to untreated controls, three consecutive PBM treatments down regulated the p21 level by 1.8-fold and upregulated Sirt1 level 1.6-fold (Fig. 8C,D). Taken together, these results suggest that the daily PBM treatments at 3 J/cm^2^ for 3 consecutive days has a lasting rejuvenating effect on aged MSCs.

**Figure 9:**
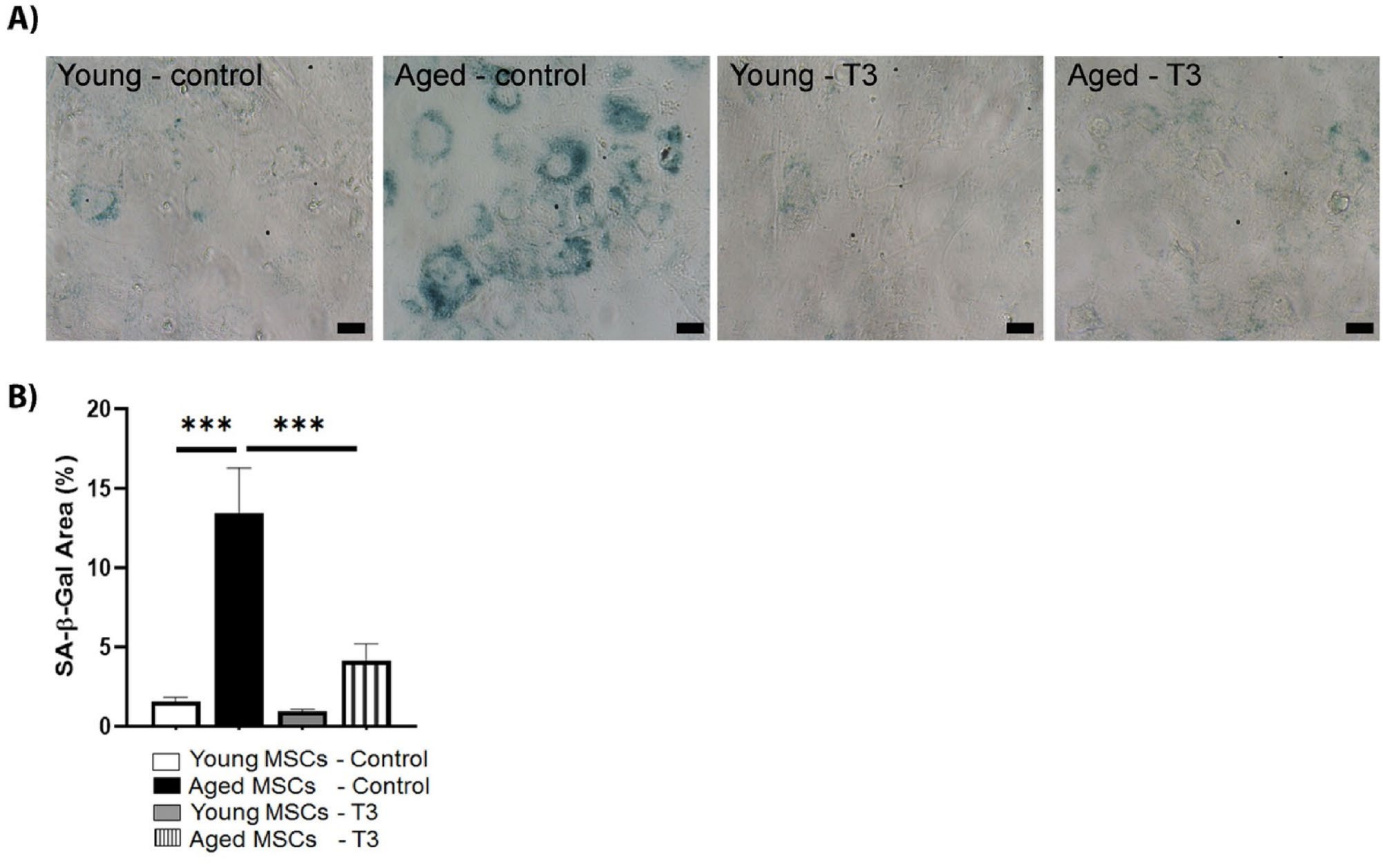
Effects of multiple doses of PBM on SA-β-Gal activity of young and aged MSCs. Young and aged MSCs underwent a PBM treatment at 3 J/cm^2^ for 3 (T3) days in a row, while untreated MSCs served as controls. On day 7, the cells were fixed and subjected to SA-β-Gal staining. (**A**) Shown are representative images of senescent cells. Scale bars: 20 μm. (**B**) Shown are mean percentages of the SA-β-Gal-stained area for each group as a bar graph. The error bars represent the standard error of mean. ***p < 0.001.

Finally, the effect of PBM on senescence-associated β-galactosidase (SA-β-Gal), a widely used biomarker for cell senescence^58^, was examined. As shown in Fig. 9A, three consecutive PBM treatments (T3) with 24-h intervals significantly inhibited the SA-β-Gal activity in aged BM-MSCs compared to their untreated counterparts (13.5% vs. 4.2%, *p* < 0.001, Fig. 9B). In fact, the reduced SA-β-Gal activity of aged BM-MSCs after three consecutive PBM treatments was comparable to that of control young MSCs. The SA-β-Gal activity of young MSCs was at the basal level and was not significantly reduced after three consecutive PBM treatments (1.56% vs. 0.98%, Fig. 9B). These results are consistent with the western blot data and further support the rejuvenating effect of PBM on aged MSCs.

### Multilineage differentiation potential of BM-MSCs

**Figure 10:**
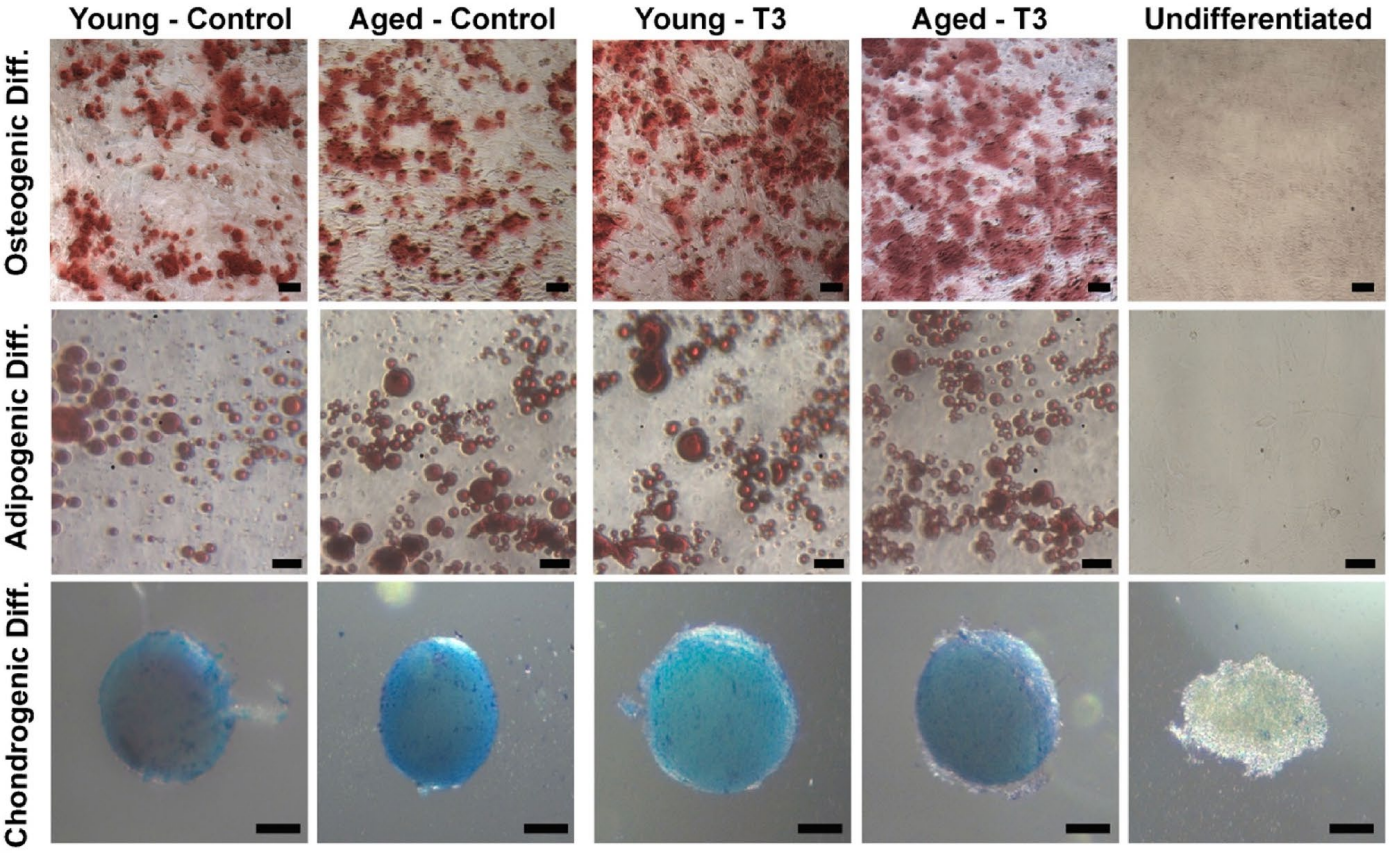
Effects of multiple doses of PBM on multi-lineage differentiation potential of young and aged MSCs: Young and aged MSCs underwent a PBM treatment at 3 J/cm^2^ for 3 (T3) days in a row while untreated MSCs served as controls. On day 7, the cells from both the control and experimental groups were harvested and subjected to differentiation experiments. Undifferentiated cells were cultured in a DMEM/F-12-based culture medium. Shown are representative images of osteogenic, adipogenic, and chondrogenic differentiation with Alizarin Red, Oil Red-O, and Alcian blue staining, respectively. In addition, representative images of undifferentiated cells stained with the same dyes are also shown. Scale bars: 50 μm.

To examine whether the multipotency of BM-MSCs is maintained after PBM treatment, MSCs were exposed to three consecutive PBM treatments (T3) as described above and were induced to differentiate into osteogenic, adipogenic, and chondrogenic lineages along with their untreated controls (Fig. 10). Alizarin Red, Oil Red-O, and Alcian Blue solutions were used to detect extracellular calcium mineralization, lipid accumulation, and proteoglycans, respectively. To confirm the specificity of the staining protocols, undifferentiated cells were also exposed to each staining protocol. As shown in Fig. 10, both PBM-treated MSCs and their untreated counterparts differentiated into all three lineages. Quantification of Alizarin Red positive cells revealed that with respect to untreated young (100% ± 8) and aged controls (132% ± 16), PBM (T3)-treated cells had a slightly higher number of osteogenic positive cells in both young (122% ± 10) and aged (155% ± 59) MSC groups (Supplementary Fig. S1). Similarly, quantification of Oil Red-O positive cells showed that compared to their untreated young (100% ± 8) and aged controls (120% ± 10), PBM (T3)-treated MSCs displayed slightly more Oil Red-O staining in both young (112% ± 16) and aged (134% ± 19) MSC groups. The differences, however, were not statistically significant (Supplementary Fig. S1). Likewise, after 3 weeks of induction in the chondrogenic differentiation medium, both PBM (T3)-treated MSCs and their untreated counterparts were able to form chondrocyte spheroids in similar size and colors (Fig. 10). Taken together, our results suggest that PBM treated MSCs retain their multi-lineage differentiation potential.

To further characterize the effect of PBM on BM-MSCs, we next examined the expression of MSC surface markers such as platelet-derived growth factor receptor alpha (PDGFRα), PDGFRβ, and Vimentin using immunofluorescence staining (Supplementary Fig. S2). The immunofluorescence staining revealed that untreated as well as PBM (T3)-treated young and aged BM-MSCs expressed the specific surface markers.

## Discussion

By characterizing BM-MSCs from young and aged mice and systematically testing their response to different modalities of PBM, the present study reveals that BM-MSCs from aged mice display a marked senescence phenotype and respond differently to the same PBM treatment than do BM-MSCs from young mice, with the beneficial effect of a single PBM treatment dissipating faster in aged MSCs than in younger ones. Nevertheless, our results also show that the beneficial effect of PBM therapy can be extended by applying three consecutive PBM treatments at 24-h intervals. These findings are of significance for improving the effectiveness of autologous stem cell therapies in older individuals who need such therapies most.

Despite considerably increased interest in stem cell research, realization of clinical applications of stem cells still remains elusive at large due to several obstacles, such as lack of xenofree clinical-grade methods for their isolation, expansion and banking, inefficient homing and integration of transplanted stem cells, and immunological issues. The latter of these obstacles can be overcome by using autologous stem cells. However, findings of several studies suggest that stem cells from older subjects are functionally compromised as a result of aging^12–14,35,59–64^ although contrasting results have also been reported^65,66^. The results of the present study are in line with the former ones and extend previous findings by showing that MSCs from older subjects express certain senescence/juvenescence markers at significantly different levels. Hence, our results together with previously published data highlight the importance of rejuvenating aged stem cells to achieve an effective autologous transplantation in older individuals.

Numerous studies have demonstrated that when applied appropriately, PBM therapy improves wound healing^67–72^, cell proliferation^30,31,73,74^, mitochondrial membrane potential and ATP production^32,73^. Based on these findings and considering the critical role of mitochondria in cell health and aging^75^, we postulated that PBM may rejuvenate aged MSCs through improving mitochondrial function. As mentioned in the introduction section, PBM in the NIR range activates mitochondria through CcO resulting in increased production of ATP^73^. However, the mechanism behind this is not well understood. It has been hypothesized that PBM causes Nitric Oxide (NO) to dissociate from CcO^26,76–78^. NO is a regulated inhibitor of CcO, which normally becomes unbound when oxygen concentrations rise^79,80^, and thus is designed to slow down the electron transport chain when oxygen is limited. Upon exposure to NIR light, NO is pushed to the unbound state, and ATP production in the mitochondria increases. This change causes NO levels to rise and, along with reactive oxygen species (ROS), is thought to contribute to the biphasic nature of PBM^34^ as these compounds are signaling molecules which can be beneficial at lower doses, but toxic at increased levels. For this reason, there is a relatively narrow power range for which PBM therapy is expected to work, with under-dosing or over-dosing being of either no benefit or of harm respectively^24,29,32^. However, the optimal power range in published studies widely vary in terms of energy density (also known as fluence) at the cellular level from 0.5 to 50 J/cm^2^ without a consensus^68,81–85^. This variation might be partially due to different types of treated tissues and cells, as well as to different light parameters such as wavelength, treatment distance and duration. While the effective fluence reported for various stem cells was more consistent and in a narrower range of 0.5 to 6 J/cm^2^ ^86^, the optimal PBM treatment strategy for aged stem cells has remained obscure, particularly when considering the narrow power range mentioned above. The present study addresses this open question through testing three different doses (i.e., 3.0, 4.5, and 6.0 J/cm^2^) at an NIR wavelength (808 nm) and shows that aged MSCs well respond to an energy density of 3 J/cm^2^. Overall, these results are in agreement with previously published data for non-aged stem cells showing that PBM treatments delivering low energy densities between 1 and 3 J/cm^2^ using an NIR wavelength have a positive effect on cell proliferation while energy densities higher than 4 J/cm^2^ remain ineffective^29,87–92^. However, our subsequent side-by-side comparison experiments revealed that the beneficial effect of a single PBM treatment fades faster (within 7 h) in aged MSCs than in younger ones, indicating that a more complex treatment regimen is needed to maintain a lasting rejuvenating effect on aged MSCs. This conclusion is also supported by an in vitro study that used cultured myotubes as a model and reported a significant response to PBM in terms of mitochondrial membrane potential and ATP synthesis between 3 and 6 h after treatment^33^.

While the short-term effects of PBM are well documented in different cell types (reviewed in^86,93,94^), strategies to prolong the beneficial effect of PBM therapies have been barely explored. Similarly, PBM studies on aged stem cells and reversing their senescence phenotype are largely missing. By testing the working hypothesis that consecutive PBM treatments at 24-h intervals induce a lasting rejuvenating effect on aged MSCs, the present study next attempted to address these needs. Indeed, compared to a single PBM treatment, two and three consecutive PBM treatments resulted in an increasingly lasting beneficial effect in terms of cell proliferation, mitochondrial respiration and ATP production, and reversal of senescence phenotype, supporting our working hypothesis. This lasting beneficial effect can be attributed to robust improvement of mitochondrial function and increased activation of secondary messenger pathways, transcription factors, and growth factor expression through PBM-induced NO and ROS signaling^24,74,93,95^. Although limited to cell proliferation and not for aged stem cells, two recent studies reported partially similar results by comparing single and multiple PBM treatments at two red (630 and 660 nm) wavelengths using fluences between 2 and 4 J/cm^2^ ^96,97^. When rat BM-MSCs were exposed to single and multiple PBM treatments at 48-h intervals for 13 days, single treatment led to a temporary increase in cell proliferation lasting for 2 days. In contrast, multiple treatments resulted in profound and long-term cell growth^96^, consistent with our results. In the second study, the positive effect of the multiple treatments on viability of stem cells from human exfoliated deciduous teeth was also significant compared to single treatment, but was less lasting (48 h)^97^. Overall, our results expand the findings of these studies and support the notion that the beneficial effect of PBM can be extended by applying different consecutive treatment strategies.

Several parameters such as wavelength, fluence, power density, pulse mode, and treatment duration seem to influence the outcome of PBM^93^. The main limitation of the present study is testing of only certain aspects of the PBM therapy for aged MSCs. As the optimization of all aforementioned parameters for aged MSCs is a daunting task and beyond the scope of a single study, we used published findings to make informed choices on selection of some parameters such as wavelength, range of fluence, and treatment strategy. For example, several studies found wavelengths between 700 and 770 nm ineffective^98–100^, whereas blue (415 nm) and green (540 nm) wavelengths were even inhibitory^91^. In contrast, red and infrared wavelengths around 660 and 810 nm respectively were generally effective^84,99,100^. We preferred an NIR wavelength (808 nm) in the present study to restore mitochondrial function—and thus to rejuvenate aged MSCs—because such a wavelength targets mitochondrial chromophores, specifically CcO, and is able to penetrate deep tissues^24,25,91,101^. We have not tested a fluence lower than 3 J/cm^2^ nor a PBM strategy involving more than 3 consecutive treatments. It is possible that fluences lower than 3 J/cm^2^ might also be effective for aged MSCs. Nevertheless, our results clearly show that a fluence at 3 J/cm^2^ is greatly effective in restoring mitochondrial function. Similarly, a PBM strategy involving 3 consecutive treatments at 24-h intervals proved to be efficient at extending the beneficial effect of the PBM therapy and reversing aging of MSCs; further improvement might be achieved by applying more complex treatment strategies in future studies.

Aging, cellular senescence and rejuvenation are complex processes associated with a wide spectrum of alterations in cell proliferation, intercellular communication, stem pool, metabolism, mitochondrial function, inflammatory cytokines, epigenetic/genetic status and so on^102–104^. To characterize the aging phenotype of MSCs and the rejuvenating effects of PBM thereon, the present study examined cell proliferation, expression of senescence and longevity-promoting markers, and mitochondrial function. Consequently, the statements made in the present study regarding aging and rejuvenation of MSCs should be considered on the basis of experimental evidence provided. Such evidence for aging includes reduced cell proliferation, increased expression of senescence markers (p16, p21, and SA-β-Gal), decreased expression of longevity-promoting markers (Sirt1 and Nrf2), and reduced mitochondrial function whereas experimental evidence supporting the rejuvenating effects of PBM includes upregulation of longevity-promoting marker Sirt1 and downregulation of senescence markers (p21 and SA-β-Gal) along with improved cell proliferation and mitochondrial function. Similar differentiation capacity exhibited by young and aged MSCs in the present study might be attributed to the complex nature of aging.

In conclusion, MSCs from older subjects display a marked senescence phenotype and require an elaborate PBM treatment compared to those from young donors. A PBM strategy consisting of three consecutive treatments at 24-h intervals with each treatment delivering a 3-J/cm^2^ energy density at an NIR wavelength improves the mitochondrial function and proliferation of aged MSCs while also reversing their senescence phenotype. These findings may have significant clinical implications in terms of improving the effectiveness of autologous stem cell therapies in older individuals.

## Supplementary information

Supplementary Figures.

## Data Availability

The manuscript includes all data generated and analyzed during this study.
