# Supplementary material for: Photobiomodulation has rejuvenating effects on aged bone marrow mesenchymal stem cells
Source: Sci Rep. 2021 Jun 22;11:13067. doi: 10.1038/s41598-021-92584-3 (PMC8219765; doi:10.1038/s41598-021-92584-3)
Supplement: Supplementary file 1 — Supplementary Figures. [file 41598_2021_92584_MOESM1_ESM.docx]

**Supplementary Information**

**Photobiomodulation has rejuvenating effects on aged bone marrow mesenchymal stem cells**

Binnur Eroglu^1, §^, Evan Genova^1, §^, Quanguang Zhang^1^, Yun Su^1^, Xingming Shi^1^, Carlos Isales^1, 2^, and Ali Eroglu^1, 3,^ *

^1^Department of Neuroscience and Regenerative Medicine, Medical College of Georgia, Augusta University; ^2^Department of Medicine, Medical College of Georgia, Augusta University; ^3^Department of Obstetrics and Gynecology, Medical College of Georgia, Augusta University, Augusta, Georgia 30912, USA

^§^These authors contributed equally and are listed alphabetically.

*Corresponding author:

Ali Eroglu, D.V.M., Ph.D.

Department of Neuroscience and Regenerative Medicine

Medical College of Georgia

Augusta University

1120 15th Street, CA-2004

Augusta, GA 30912, USA

E-mail: aeroglu@augusta.edu

Tel: (706) 721-7595

Fax: (706) 721-0340

Keywords: Photobiomodulation, low-level laser therapy, stem cells, mitochondria, aging, senescence, rejuvenation

Short Title: Photobiomodulation rejuvenates stem cells

**Supplementary Figure S1: Comparison of multilineage differentiation potential of young and aged MSCs: (A)** Quantification of Alizarin Red positive cells.  **(B)** Quantification of Oil Red-O positive cells. Both Alizarin and Oil Red-O stained areas (%) were calculated by ImageJ software. The values were normalized to the untreated young control and expressed as a relative percentage. The PBM treatments induced insignificant increases in osteogenic and adipogenic differentiation compared to the untreated controls.


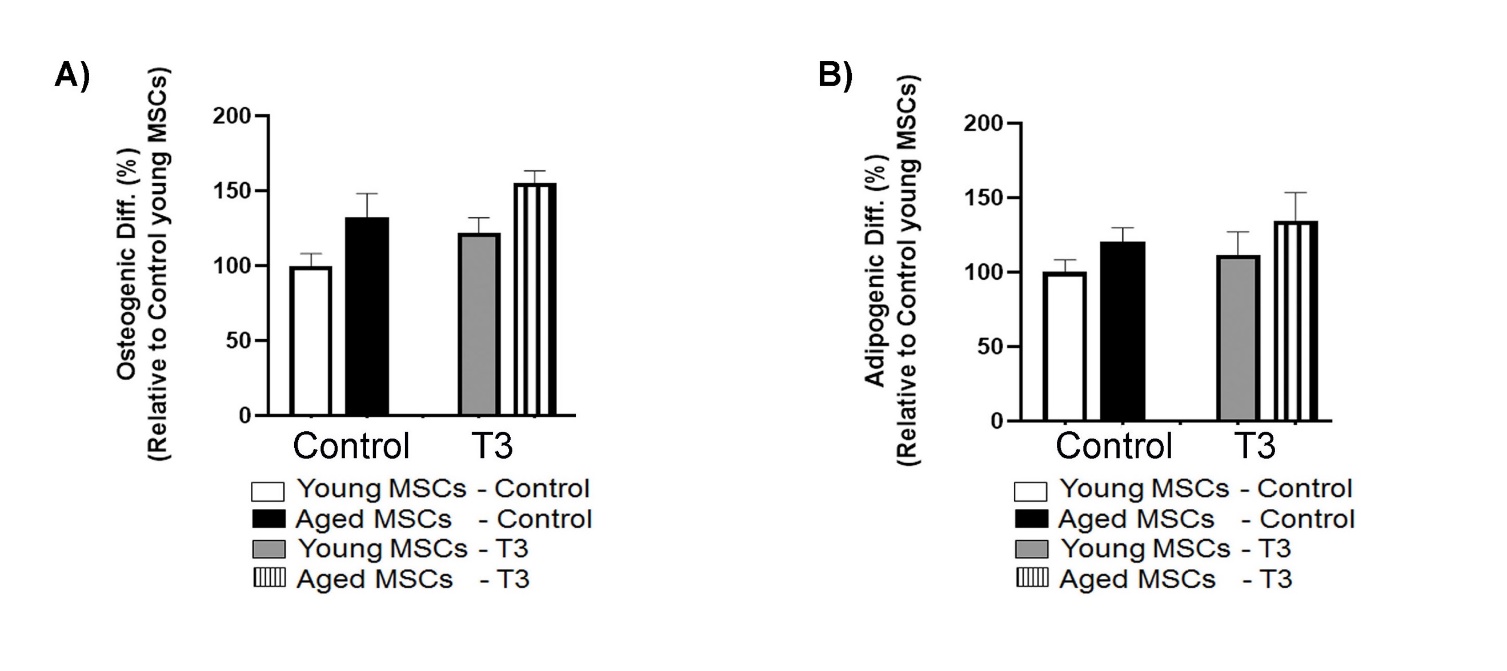


**Supplementary Figure S2: Effects of multiple doses of PBM on BM-MSC specific surface markers:** **(A)** Shown are representative bright field images of BM-MSCs. Scale bars: 50μm. **(B),** **(C),** and **(D)** Shown are representative immunofluorescence images of the MSC surface markers of PDGFRα (red), PDGFRβ (red), and Vimentin (red), respectively, whereas cell nuclei are in blue. Scale bars: 20μm.


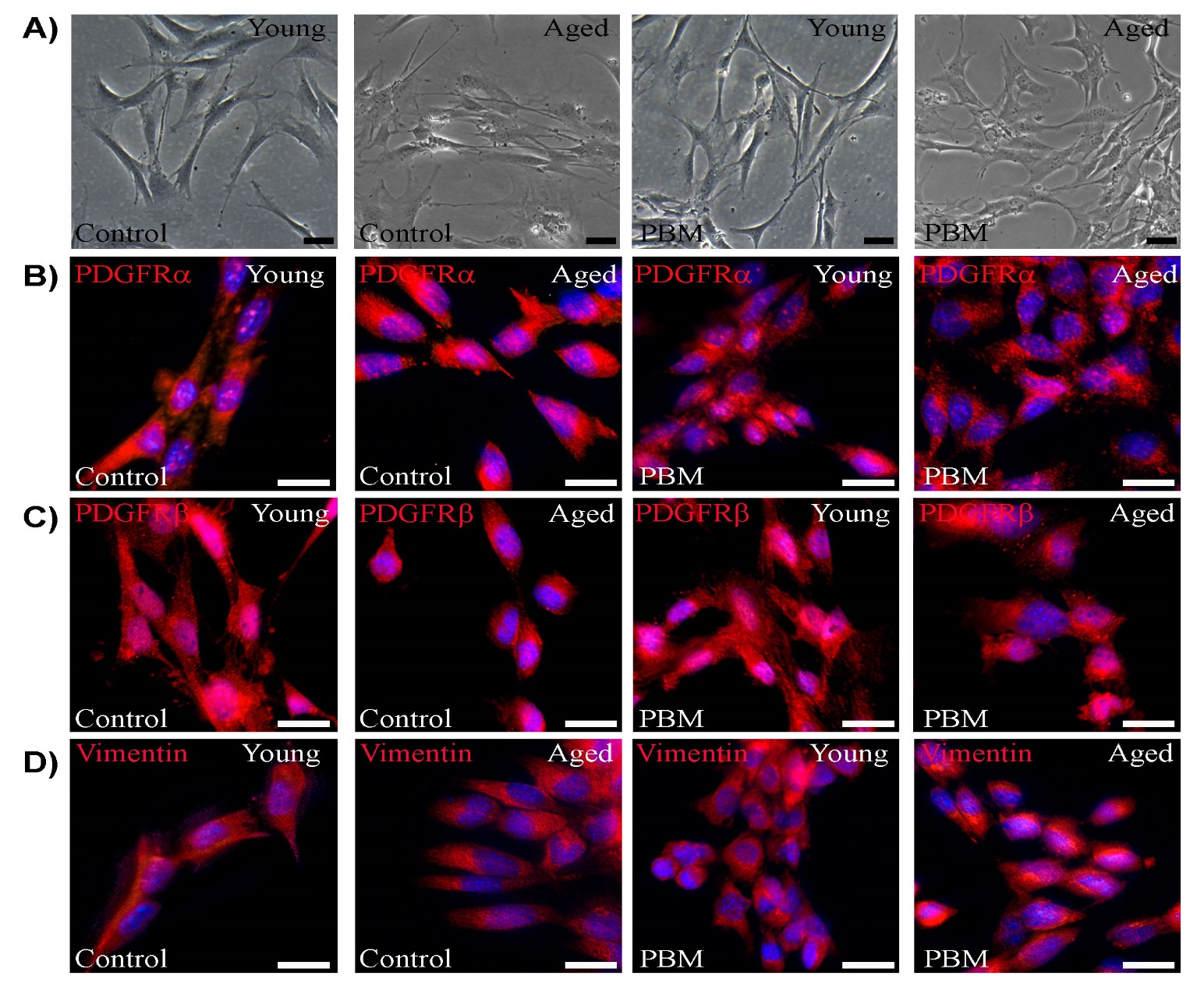


**Figure S3:** Uncropped western blot images of Figure 2 presented in the main text.

**
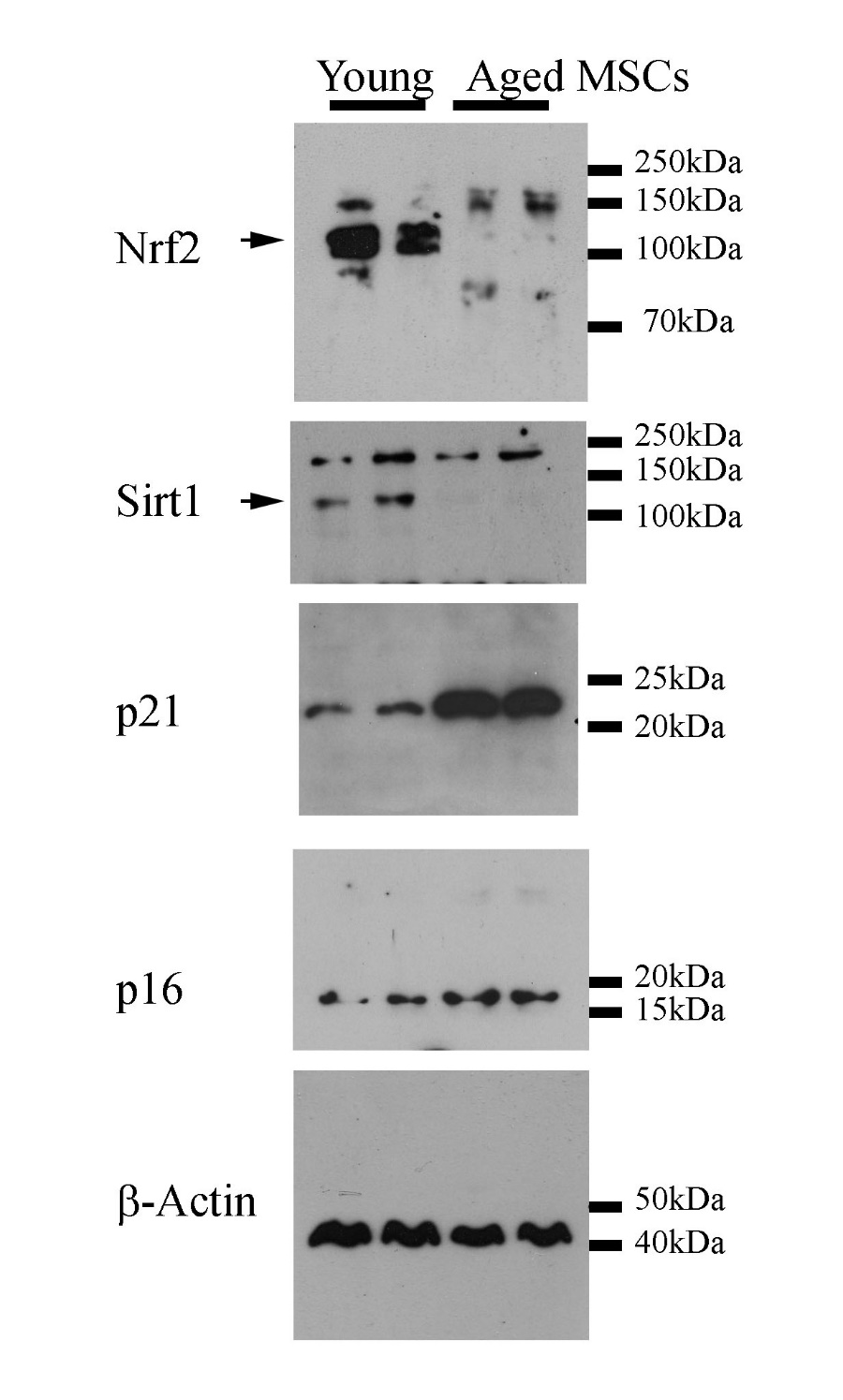
**

**Figure S4:** Uncropped western blot images of Figure 8C presented in the main text.

**
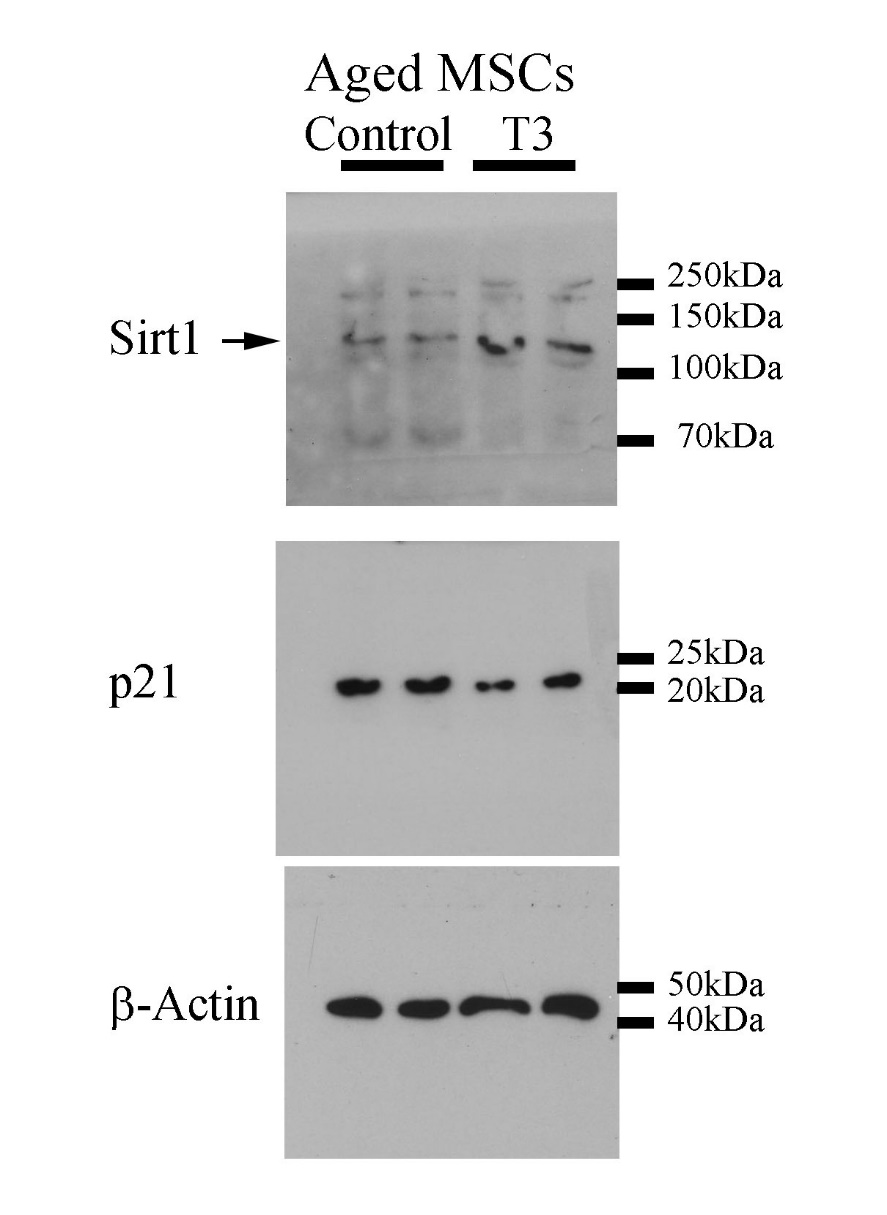
**
